# The Effects of Cold Arm Width and Metal Deposition on the Performance of a U-Beam Electrothermal MEMS Microgripper for Biomedical Applications

**DOI:** 10.3390/mi10030167

**Published:** 2019-02-28

**Authors:** Marija Cauchi, Ivan Grech, Bertram Mallia, Pierluigi Mollicone, Nicholas Sammut

**Affiliations:** 1 Department of Mechanical Engineering, Faculty of Engineering, University of Malta, MSD 2080 Msida, Malta; pierluigi.mollicone@um.edu.mt; 2 Department of Microelectronics and Nanoelectronics, Faculty of Information and Communication Technology, University of Malta, MSD 2080 Msida, Malta; ivan.grech@um.edu.mt (I.G.); nicholas.sammut@um.edu.mt (N.S.); 3 Department of Metallurgy and Materials Engineering, Faculty of Engineering, University of Malta, MSD 2080 Msida, Malta; bertram.mallia@um.edu.mt

**Keywords:** microelectromechanical systems (MEMS), microgrippers, electrothermal actuation, red blood cells, micromanipulation, SOIMUMPs™, single crystal silicon, cold arm width, metal deposition

## Abstract

Microelectromechanical systems (MEMS) have established themselves within various fields dominated by high-precision micromanipulation, with the most distinguished sectors being the microassembly, micromanufacturing and biomedical ones. This paper presents a horizontal electrothermally actuated ‘hot and cold arm’ microgripper design to be used for the deformability study of human red blood cells (RBCs). In this study, the width and layer composition of the cold arm are varied to investigate the effects of dimensional and material variation of the cold arm on the resulting temperature distribution, and ultimately on the achieved lateral displacement at the microgripper arm tips. The cold arm widths investigated are 14 μm, 30 μm, 55 μm, 70 μm and 100 μm. A gold layer with a thin chromium adhesion promoter layer is deposited on the top surface of each of these cold arms to study its effect on the performance of the microgripper. The resultant ten microgripper design variants are fabricated using a commercially available MEMS fabrication technology known as a silicon-on-insulator multi-user MEMS process (SOIMUMPs)™. This process results in an overhanging 25 μm thick single crystal silicon microgripper structure having a low aspect ratio (width:thickness) value compared to surface micromachined structures where structural thicknesses are of the order of 2 μm. Finite element analysis was used to numerically model the microgripper structures and coupled electrothermomechanical simulations were implemented in CoventorWare${}^{®}$. The numerical simulations took into account the temperature dependency of the coefficient of thermal expansion, the thermal conductivity and the electrical conductivity properties in order to achieve more reliable results. The fabricated microgrippers were actuated under atmospheric pressure and the experimental results achieved through optical microscopy studies conformed with those predicted by the numerical models. The gap opening and the temperature rise at the cell gripping zone were also compared for the different microgripper structures in this work, with the aim of identifying an optimal microgripper design for the deformability characterisation of RBCs.

## 1. Introduction

Microgrippers are an example of microelectromechanical systems (MEMS) that are extensively applied in fields dealing with the handling, positioning and assembly of micromechanical parts [1,2], as well as with the safe manipulation and characterisation of biological tissues and cells [3,4,5,6,7]. These working environments make studies on the performance of MEMS microgrippers essential. Previous works on MEMS microgrippers have focused on the optimisation of a number of factors including their design and kinematic structure [8,9], the structural materials used [10,11], the actuation and sensing mechanisms [12], the operational requirements and limitations [13], the fabrication process used [14,15,16,17], and the environment in which they will be operated [18].

This paper focuses on a horizontal electrothermally actuated ‘hot and cold arm’ MEMS microgripper design developed for the manipulation and deformability study of human red blood cells (RBCs). Thermally activated beam flexure has been one of the leading actuation principles within the MEMS domain [19,20], with this mostly being due to the advantages of a simple structure that can be easily fabricated and that can achieve large output forces with low driving voltages. The authors’ previous work [21] presented a number of essential design and fabrication considerations for the reliable performance of such a microgripper design. The studied failure mechanisms were associated with surface micromachined MEMS microgrippers fabricated with PolyMUMPs™ and having a structural thickness of the order of 2 μm. These mechanisms included out-of-plane buckling of the hot arm, fracture of the arms due to the build-up of stress concentrations, residual stresses, and device stiction. This paper presents the same microgripper design but this time based on a different commercially available manufacturing process—a silicon-on-insulator (SOI) micromachining process known as SOIMUMPs™—that results in a free-standing microgripper structure with a much lower aspect ratio (width:thickness) value due to its inherently larger structural thickness (25 μm). Such dimensional characteristics help to mitigate the microgripper’s malfunction from failure mechanisms associated with surface micromachined microgripper structures having higher aspect ratios. Additionally, in another study [16], the authors have analytically and numerically compared the performance of microgripper structures fabricated with the PolyMUMPs™ and SOIMUMPs™ processes. However, the mentioned study lacked experimental data for the SOIMUMPs™ structures, which is now being presented in the current work. In this regard, the motivation behind the current study is to investigate the feasibility of a commercially available bulk micromachining technology—SOIMUMPs™—to fabricate a traditional ‘hot and cold arm’ microgripper design and to improve its performance with respect to a surface micromachined microgripper fabricated with PolyMUMPs™. This study aims to optimise the electrothermomechanical performance of the fabricated microgripper structure based on the requirements to micromanipulate and study the deformability properties of RBCs, and focuses on obtaining a sufficient opening stroke of the microgripper arms and a minimum temperature rise at the cell gripping zone.

**Figure 1:**
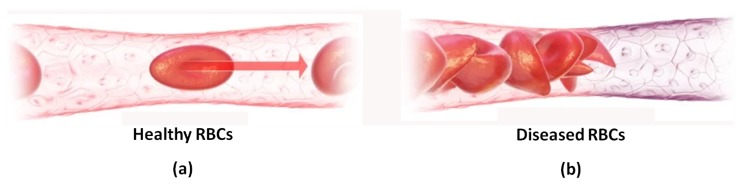
The flow of red blood cells (RBCs) through a normal capillary with: (**a**) unrestricted blood flow in the case of healthy cells, and (**b**) with diseased RBCs blocking the blood flow due to their inability to undergo the required cell deformation [25].

The presented microgripper design is aimed to study the deformability characteristics of RBCs whose pathophysiological relevance allows them to serve as an important marker of the health status of patients [22,23]. RBCs are highly flexible bi-concave discs with an approximate diameter of 8 μm and an approximate thickness of 2 μm that can be reliably tested under atmospheric conditions through the use of adequate laboratory procedures [24]. The main role of RBCs is to carry both oxygen and carbon dioxide (CO${}_{2}$) between the lungs and the body tissues. While the healthy RBCs are able to deform and freely flow even through capillaries with smaller diameters supplying oxygen as necessary, this is not the case for situations where certain diseases such as diabetes, sickle cell anemia and malaria are present. Such conditions alter the mechanical properties of RBC membrane structures, with the result that the RBCs cannot deform to flow through the small blood vessels and thus they cannot deliver the much needed oxygen to the biological tissues (Figure 1). The expected difference in the deformability properties of healthy and diseased RBCs allows the designed microgripper to be used as a diagnostic tool for the identification of the disease status of patients. In order to achieve this, the presented microgripper design will need to be enhanced in the authors’ future work to include force feedback that will enable both contact detection between the arm tips and the cell, as well as the attainment of gripping force measurements. The grasping force exerted by the microgripper arm tips on the gripped RBC is expected to increase to achieve the same cell deformation in diseased cells due to their larger membrane stiffness. Contact detection is essential to protect both the microgripper arms and the cell from damage while the gripping force feedback will help to define important cell membrane parameters and to compare the obtained gripping force profiles for the different cells.

## 2. The ‘Hot and Cold Arm’ Microgripper Design

**Figure 2:**
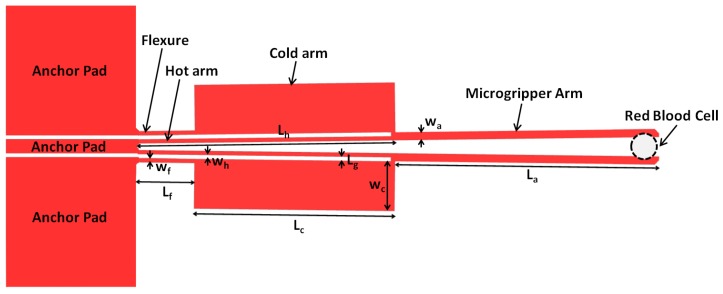
Schematic diagram of an actuated U-shape ‘hot and cold arm’ microgripper design gripping a red blood cell at the microgripper arm tips.

This work presents a number of microgripper structures whose design is based on the U-shape ‘hot and cold arm’ electrothermal actuator configuration [26]. Such a configuration is shown in Figure 2, with each actuator consisting of two parallel arms with different widths, the narrow hot arm and the wide cold arm, which experience unequal resistive heating resulting in asymmetric thermal expansion under an applied potential. The presence of the flexure component then permits the bending movement of the actuator while the microgripper arms create an amplified lateral displacement achieved at the cell gripping zone defined by the arm tips. This motion drives the two microgripper arms to open and to then grip the required object at the arm tips upon closing [27].

One of the main factors that affects the performance of the presented microgripper design is the resulting temperature difference between the hot and cold arms under an actuation voltage. The larger this temperature difference, the more efficient a horizontal microgripper is expected to be. A higher efficiency of a horizontal microgripper is defined by a larger in-plane displacement obtained at the microgripper arm tips for the same applied voltage. A number of studies [28,29,30] have shown that one way to maximise the temperature difference between the hot and cold arms is by designing a two-hot-arm thermal actuator which results in the electrical current completely bypassing the cold arm. The current study, however, considers a traditional horizontal thermal actuator consisting of one hot arm and one cold arm, and attempts to optimise this configuration as a continuation of the authors’ previous works [16,21]. The ratio of the flexure length to the hot arm length has already been optimised in the authors’ previous work [26], with the optimised value of 0.23 implemented in the current work (as given in Table 1, $L_{f}$ = 46 μm, $L_{h}$ = 200 μm, $L_{f}$/$L_{h}$ = 0.23). Moreover, the flexure is generally manufactured with the same width as the hot arm, which is in turn usually designed with the smallest dimension that the fabrication process is capable of achieving (3 μm in the case of the SOIMUMPs™ process). The thickness of the structure is also dependent on the fabrication process (25 μm in the case of the SOIMUMPs™ process). These considerations have resulted in the identification of the cold arm width as a potential unstudied parameter for optimisation in this work. A number of microgripper design variants with five different cold arm widths covering a wide range of possible values (14 μm–100 μm) for this type of microgripper structure, as well as a different layer composition of the cold arm are thus presented in this study in order to investigate the effect of these parameters on the performance of the traditional electrothermal microgripper. The geometrical dimensions of the designed microgrippers with five cold arm width variants are given in Table 1. The layer composition of the cold arm is varied by depositing a gold metal layer with a thin chromium adhesion layer on top of the single crystal silicon (SCS) structure. These variations result in ten microgripper design variants (five cold arm widths with and without a gold layer on the cold arm).

## 3. SOIMUMPs™ Fabrication Technology

**Figure 3:**
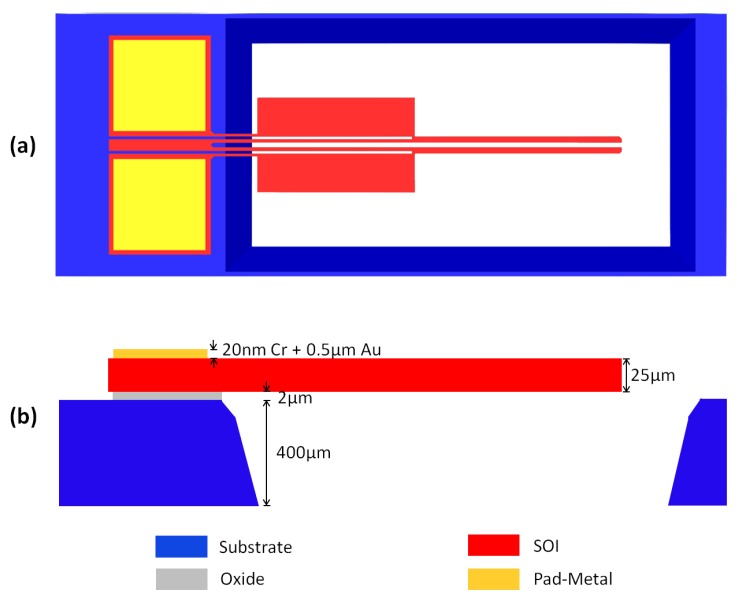
A microgripper structure fabricated with the silicon-on-insulator multi-user MEMS process (SOIMUMPs)™ with shown: (**a**) plan, and (**b**) cross-sectional views (not-to-scale).

The microgripper test structures presented in this work were fabricated by MEMSCAP, Inc. (Durham, NC, USA), using one of their standard and commercially available Multi-User MEMS Processes (MUMPs${}^{®}$), known as SOIMUMPs™. The SOIMUMPs™ fabrication technology [31] is an SOI micromachining process that results in an overhanging 25 μm thick SCS structure that is identified as the SOI layer in the process. The SCS structural layer is attached to a 400 μm thick (1 0 0) silicon handle wafer, known as the Substrate in the process, with a 2 μm thick oxide layer that is buried in between (Figure 3). Patterning of the SOI device layer and the silicon handle wafer are achieved by photolitography and they are subsequently deep reactive ion etched down to the buried oxide layer. The buried oxide layer is then removed using a vapour etch, resulting in an overhanging movable structure. A metal stack, known as the Pad-Metal layer in the process, consists of a 20 nm thick chromium adhesion layer and a 500 nm thick gold layer, and can be deposited over the SCS structure by electron beam physical vapour deposition for bonding, probing or electrical routing purposes.

## 4. Numerical Model

**Figure 4:**
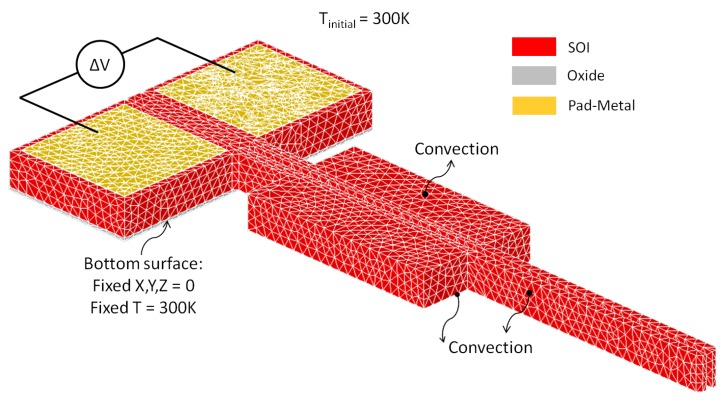
The SOIMUMPs™ electrothermal microgripper model showing the implemented parabolic tetrahedral mesh and the applied electrical, thermal and mechanical boundary conditions.

A numerical model based on the finite element method (FEM) was developed in CoventorWare${}^{®}$ (Coventor, Inc., Cary, NC, USA) and steady-state electrothermal and thermomechanical simulations were sequentially carried out to study the performance of the designed microgrippers. The discretised three-dimensional (3D) model of one of the microgripper structures fabricated with the SOIMUMPs™ process is shown in Figure 4.

A number of electrical, thermal and mechanical boundary conditions were implemented within the numerical model, as also shown in Figure 4. The actuation voltage was applied across the metallised anchor pads whose temperature was also fixed to room temperature, and whose motion was restricted in all directions throughout the analyses. Room temperature was also applied as an inital temperature condition to all the model elements. Moreover, a convective heat transfer coefficient was equally applied to the microgripper surfaces in order to simulate heat losses by natural convection in air.

A critical aspect in the modelling of electrothermally actuated structures is accounting for the temperature dependency of certain material properties. Table 2 compares the measured resistance and dissipated power values for one of the fabricated microgripper structures, to the values obtained from the FEM model assuming temperature-independent electrical conductivity properties. It can be observed that the difference between the measured and the simulated values increases rapidly as the voltage increases. This is due to the wrong assumption of applying temperature-invariant electrical conductivity properties within the model. The higher temperatures developing at higher voltages significantly alter the material’s conductivity, resulting in errors as high as 52% and 34% in the resistance and power dissipation values respectively for SOIMUMPs™ microgripper structures (Table 2).

**Figure 5:**
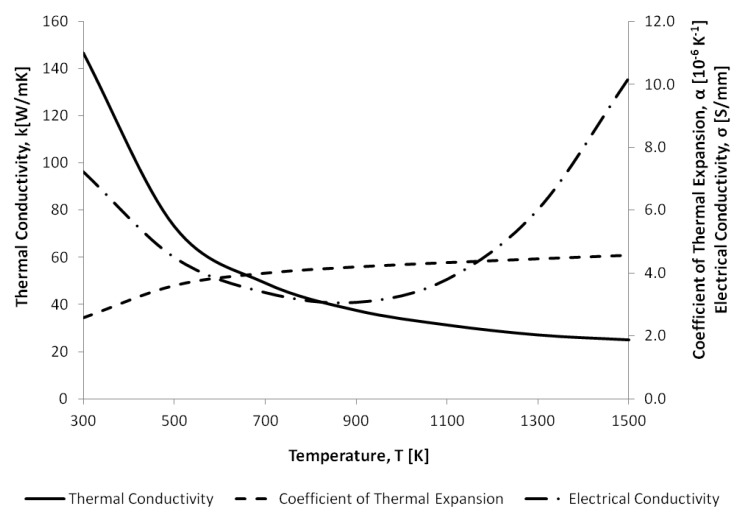
Variation of the thermal conductivity, the coefficient of thermal expansion and the electrical conductivity of single crystal silicon (SCS) with temperature [34].

**Figure 6:**
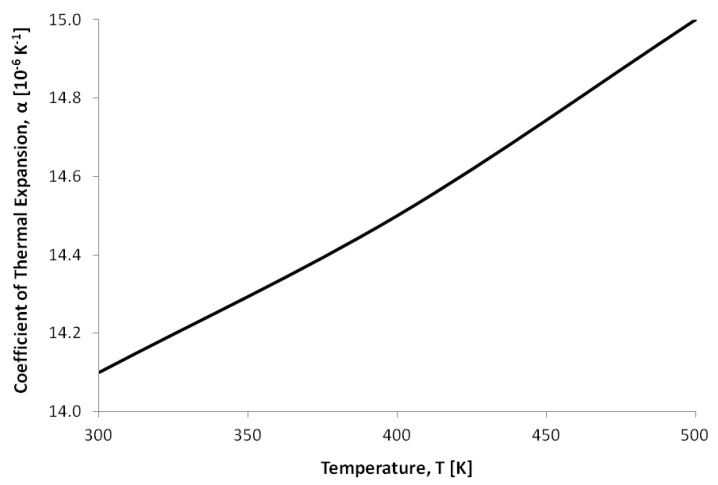
Variation of the coefficient of thermal expansion with temperature for the Pad-Metal layer (chromium + gold) defined in the SOIMUMPs™ process [31].

For this reason, temperature-dependent material properties were used within the microgripper models in CoventorWare${}^{®}$. This work modelled the temperature dependency of the thermal conductivity, the coefficient of thermal expansion and the electrical conductivity of SCS, as well as the coefficient of thermal expansion of the metal layer, as given in Table 3, and in Figure 5 and Figure 6, respectively. For semiconductors such as SCS, the electrical conductivity property is influenced by the carrier concentration and the carrier mobility, both of which are strong functions of the doping profile and the temperatures developed, respectively. Moreover, the anisotropic nature of SCS results in direction-dependent elastic and mechanical properties. The crystalline structure of SCS exhibits cubic symmetry, making it possible to define its elastic and mechanical properties with orthotropic material constants. The latter are given in the frame of reference of a standard (100) silicon wafer in Table 3, with the (100) plane being the typical silicon wafer orientation featuring in MEMS microfabrication processes [32,33].

## 5. Experimental Setup

**Figure 7:**
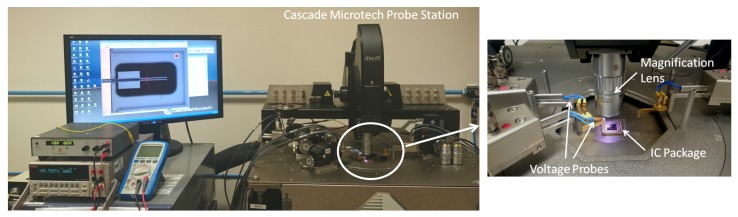
Setup for the experimental characterisation of the fabricated microgripper structures on the Cascade Microtech probe station. The microgripper structures were mounted on an integrated circuit (IC) package and actuated through the use of voltage probes.

Experimental testing was performed on the ten microgripper design variants using the setup shown in Figure 7. Electrical actuation of the microgripper structures was achieved under atmospheric pressure on a Cascade Microtech Summit 11,000/12,000 B-series probe station (Cascade Microtech, Inc., Beaverton, OR, USA) through the use of voltage probes having an in-built *x*-*y*-*z* positioning system. A power supply was used to apply a potential in increments of 0.5 V up to 12 V across the probe pads of each fabricated microgripper structure. The resulting gap opening between the microgripper arm tips was then characterised through the use of the optical microscope-based vision system embedded within the probe station.

## 6. Results and Discussion

The electrothermomechanical performance of the ten microgripper design variants has been studied both numerically and experimentally in this work. A lumped analytical model for the SOIMUMPs™ microgripper structure has already been developed and presented in the authors’ previous work [16] where the temperature distribution and tip displacement results were shown to be in good agreement with the developed numerical model.

### 6.1. Thermal Analyses

The temperature distribution developed within a microgripper structure during actuation is an important design criterion as it significantly influences the microgripper’s performance. A larger temperature difference developed between the hot and cold arms is expected to result in an increase in the gap opening achieved between the microgripper arm tips due to a larger difference between the thermal expansion of the two arms. Another important aspect of an electrothermal microgripper designed for the handling of biological cells is the temperature developed at the cell gripping zone.

**Figure 8:**
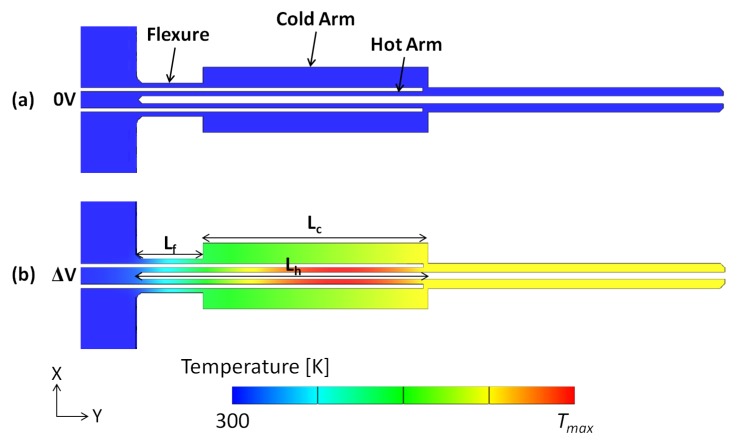
Simulated steady-state temperature distribution plot of a SCS microgripper structure fabricated with SOIMUMPs™: (**a**) without an applied potential, and (**b**) with an applied potential.

**Figure 9:**
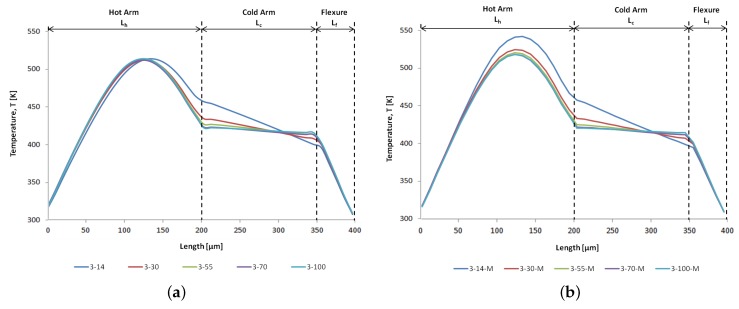
Comparison of the numerical results for the temperature profiles along the different actuator components at 12 V for the microgripper designs: (**a**) without metal deposition on the cold arm, and (**b**) with metal deposition on the cold arm. The ‘3-14’ nomenclature respresents the microgripper structure with a hot arm width of 3 μm and a cold arm width of 14 μm, while the ‘3-14-M’ nomenclature represents the microgripper structure with the same dimensions as the ‘3-14’ one but with metal deposited on the cold arm.

Thermal characterisation of the microgripper as a function of the applied voltage has not been conducted experimentally in this study due to the absence of the necessary equipment to obtain such data. A micro radiometric thermal imaging microscope with adequate temperature and spatial resolutions would be able to measure and display the temperature distribution over the microgripper’s surface, enabling the quick detection of hot spots and thermal gradients, as well as the possibility of numerical benchmarking. In the absence of such experimental data, the current work presents and compares the numerical thermal characterisation of the different microgripper designs under electrothermal actuation. The predicted temperature distribution on an actuated SOIMUMPs™ microgripper structure, as obtained with CoventorWare${}^{®}$, is shown in Figure 8. Figure 9 then compares the numerical results for the temperature distribution along the hot arm, cold arm and flexure components of the considered microgripper design variants. It can be observed that the maximum temperature is, in all the cases, located on the hot arm, independent of the cold arm width and the presence of metal deposition on the cold arm. This is an inherent characteristic of the ‘hot and cold arm’ actuator design. Another interesting observation made from Figure 8 is that the arm tips of the actuated SOIMUMPs™ microgripper structure undergo quite a significant rise in temperature with respect to room temperature (300 K). The maximum temperature rise developed at the microgripper arm tips should generally be limited to around 100 K to ensure the safe manipulation of living cells and tissues. The higher thermal conductivity of SCS when compared to that of polycrystalline silicon thus poses a limitation in this regard for microgripper structures fabricated with the SOIMUMPs™ process as opposed to the PolyMUMPs™ process as discussed in the authors’ previous work [16], and as will be further discussed in Section 6.2 in relation to the gap opening at the microgripper arm tips.

It can be noted that the value of the maximum temperature on the hot arm is the same for all the microgripper designs shown in Figure 9a, i.e., the temperature of the hot arm is not influenced by the cold arm width in this case. This is as expected given that the hot arm width, which determines the maximum temperature reached in a standard (i.e., composed of SCS only) ‘hot and cold arm’ microgripper design, is 3 μm for all the designs. This is, however, not the case for the set of microgripper designs that have metal deposited on the cold arm (Figure 9b). It can be observed that, although the hot arm width is the same (3 μm) for all the structures even in this case, as the cold arm width increases, the maximum temperature developed on the hot arm changes. This behaviour can be attributed to the fact that the presence of metal deposition on just the cold arm creates a further asymmetry (in addition to a dimensional one) between the hot and cold arms. This results in a decrease in the overall resistance of the structure as well as a decrease in the current density of the hot arm as the cold arm with deposited metal increases in width.

The process of adding a metal layer on the cold arm affects the temperature distribution on the hot arm as can be seen by comparing the design variants without and with metal deposition on the cold arm (e.g., 3-14 and 3-14-M) in Figure 9a,b, respectively. The maximum temperature developed on the hot arm increases due to the lowered resistance of the structure caused by the metal layer deposited on the cold arm, and the resulting increase in current and in current density within the hot arm. The temperature distributions on the cold arm and the flexure remain unaffected. Another observation is that irrespective of the presence of metal deposition on the cold arm, the temperature distribution along the cold arm varies with cold arm width, with the average temperature on the cold arm and the temperature gradient along the cold arm length decreasing with increasing cold arm width. The increasing cold arm width reduces the overall resistance of the microgripper, resulting in less Joule heating.

In the ‘hot and cold arm’ microgripper design, the lateral displacement achieved at the microgripper arm tips is influenced by the temperature difference developed between the hot and cold arms, with this displacement expected to increase as the stated temperature difference increases for the same applied voltage. Different ways to maximise this temperature difference include designing the hot arm with the smallest possible cross-sectional area as allowed by the capability of the fabrication process (the resulting effect is to maximise the temperature on the hot arm due to a higher current density within the hot arm), increasing the width of the cold arm (the resulting effect is to minimise the temperature on the cold arm due to a decrease in the resistance of the cold arm), and depositing a metal layer on the cold arm (the resulting effect is to maximise the temperature on the hot arm due to an asymmetric metal deposition on the hot and cold arms and a higher current density within the hot arm). The SOIMUMPs™ fabrication technology recommends a minimum feature width of 3 μm to be used within the process [31], and this value has in fact been used for the hot arm width for all the microgripper designs in this work.

**Figure 10:**
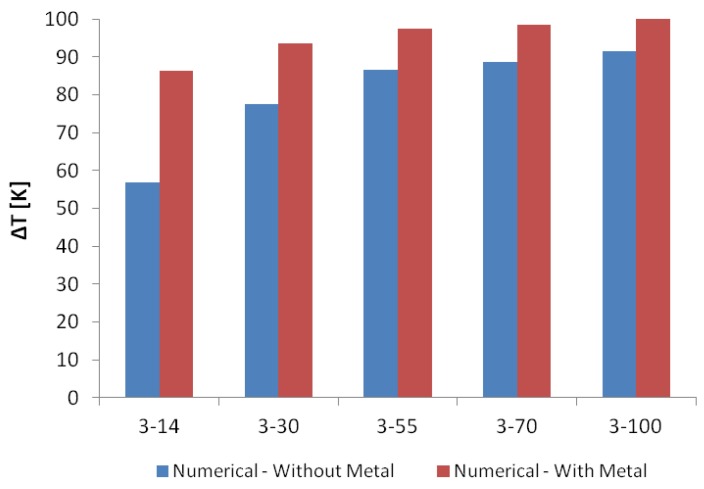
Comparison of the resulting numerical temperature difference between the hot and cold arms for the considered microgripper designs at 12 V.

The resulting numerical temperature difference between the hot and cold arms is shown in Figure 10. It can be observed that for the design variants without (blue) and with (red) metal deposition, the temperature difference increases both with an increase in the cold arm width, as well as by adding a metal layer on the cold arm. The increasing cold arm width results in a decrease in the resistance of the cold arm, lowering the temperature distribution achieved on the cold arm and thus increasing the temperature difference between the hot and cold arms. It can, however, be noted that the benefit of depositing metal on the cold arm to maximise the resulting temperature difference decreases as the cold arm width increases. While the temperature difference in the case of the structures without metal deposition increases by 61% from a cold arm width of 14 μm to a cold arm width of 100 μm, the same temperature difference increases by only 16% for the same range of cold arm widths in case of the structures with the deposited metal layer on the cold arm. The smaller increase in the temperature difference percentage in the case of the structures with deposited metal layer on the cold arm is due to the fact that the combination of increasing the width of the cold arm while the latter has metal deposited on it causes a decrease in the maximum temperature of the hot arm (as opposed to a constant maximum hot arm temperature in the case of the structures without metal deposition) in addition to a decrease in the temperature of the cold arm (Figure 9b), consequently resulting in a much smaller effective temperature difference between the hot and cold arms.

### 6.2. Structural Analyses

**Figure 11:**
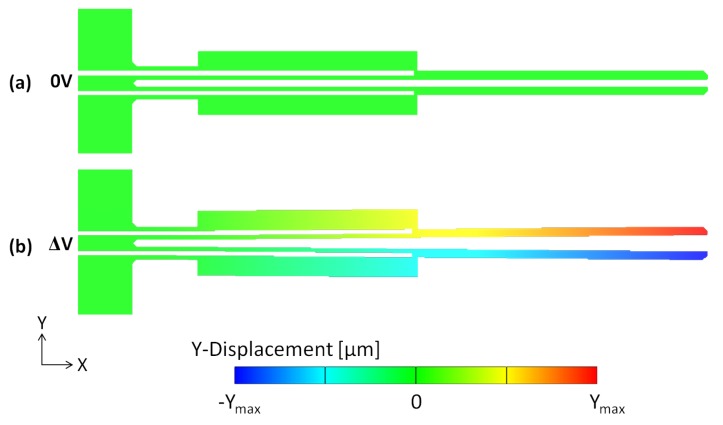
Simulated steady-state lateral displacement plot of a SCS microgripper structure fabricated with SOIMUMPs™: (**a**) without an applied potential, and (**b**) with an applied potential. The maximum *y*-displacement of the actuated microgripper arms is located at the cell gripping zone.

**Figure 12:**
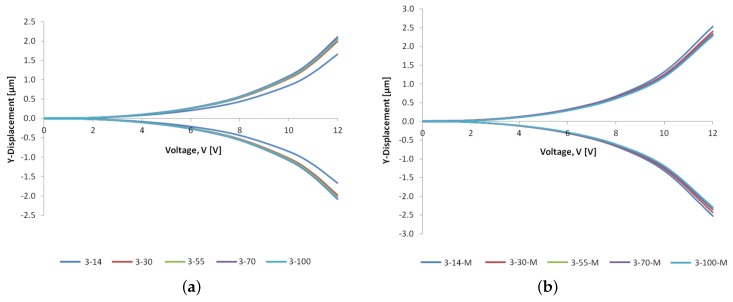
Comparison of the numerical results for the *y*-displacement of each microgripper arm as a function of voltage for the microgripper designs: (**a**) without metal deposition on the cold arm, and (**b**) with metal deposition on the cold arm.

The design of a horizontal MEMS microgripper should be such that it maximises the achieved lateral end-effector displacement as required for the application in consideration, while minimising the temperature developed at the arm tips in the case of biomedical applications. The predicted lateral displacement distribution of the designed microgripper structure is shown in Figure 11. The steady-state in-plane displacement profiles are given as a function of voltage for all the design variants in Figure 12. It can be observed from Figure 12 that the obtained total gap opening of the design variants is larger in the presence of metal deposition on the cold arm. Moreover, the opening displacement of each arm increases with increasing cold arm width in the case of the structures without metal deposition while it remains approximately the same in the case of the structures having the deposited metal layer on the cold arm (Figure 12). This outcome is in line with the resulting variation in the effective temperature difference with cold arm width observed in the case of the structures with and without metal deposition as outlined in Section 6.1.

**Figure 13:**
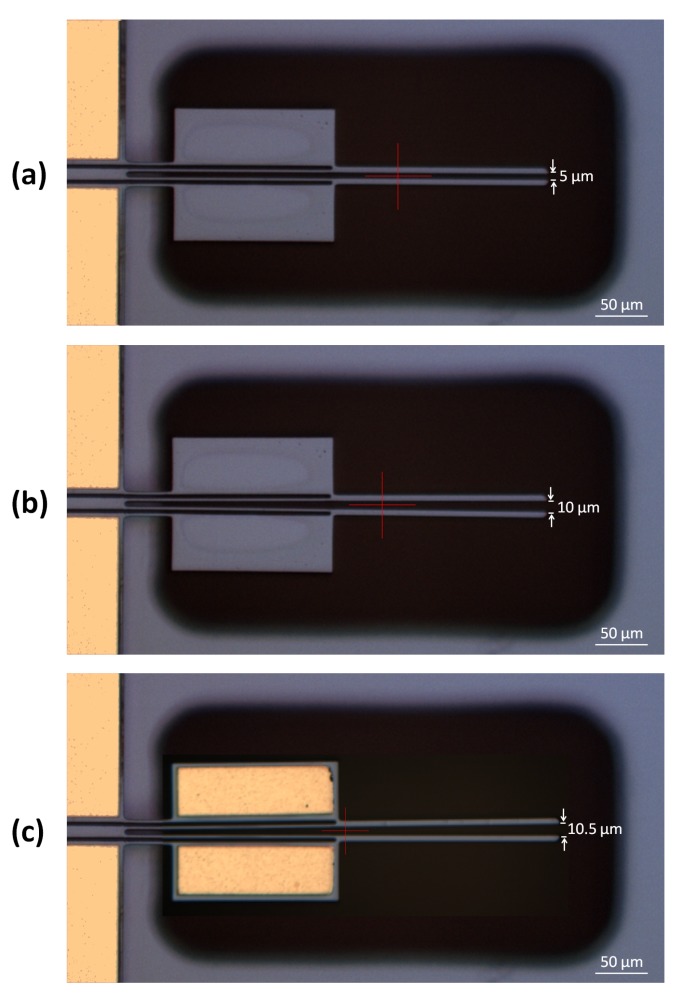
Optical microscopy images of one of the fabricated microgripper structures (hot arm width = 3 μm, cold arm width = 55 μm): (**a**) when not actuated, (**b**) when electrothermally actuated with 12 V, and (**c**) when the same microgripper structure, however this time with metal deposition on the cold arm, is electrothermally actuated with 12 V.

**Figure 14:**
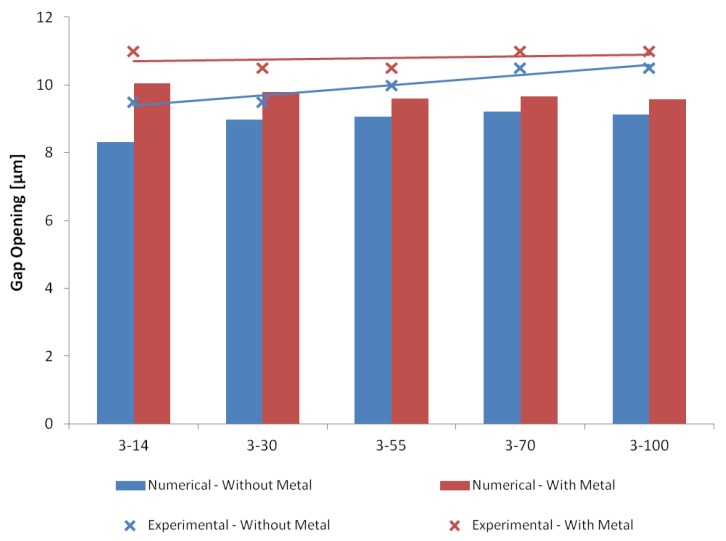
Experimental and numerical results for the total gap opening of the considered microgripper designs (with and without metal deposition on the cold arm) at 12 V. All designs start from a gap opening of 5 μm which is the gap opening in the closed position.

The numerical models of the ten microgripper design variants were validated through experimental tests performed under atmospheric pressure using the setup described in Section 5. Optical microscopy images of the microgripper structure with a cold arm width of 55 μm in the closed (i.e., not actuated) and actuated positions, as well as of the microgripper structure with the same cold arm width and with metal deposition on the cold arm in the actuated position, are shown in Figure 13. All microgripper structures have a gap opening of 5 μm when not actuated. Figure 14 gives a comparison of the simulated and experimental measurement results of the total gap opening at the microgripper arm tips for all the design variants. It is demonstrated that the results predicted by the numerical models are well in line with the experimental results, with an average discrepancy in the region of 10%. Moreover, both measured and simulated gap openings at 12 V increase with cold arm width for the design variants without metal deposition on the cold arm, while they remain approximately the same for the design variants with metal deposition on the cold arm. The measured gap opening values confirm that all design variants can handle an RBC with an approximate diameter of 8 μm.

**Figure 15:**
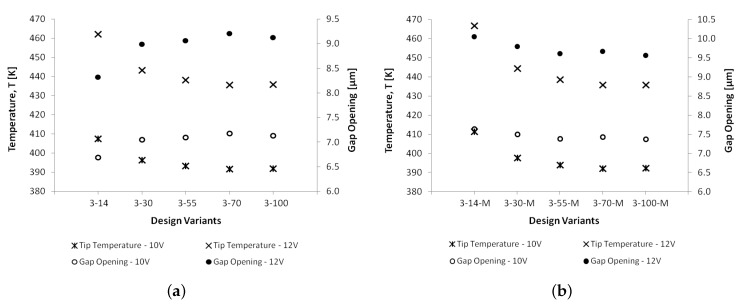
Comparison of the numerical results for the temperature and the total gap opening at the microgripper arm tips at actuation voltages of 10 V and 12 V for the considered microgripper designs: (**a**) without metal deposition on the cold arm, and (**b**) with metal deposition on the cold arm.

As already highlighted in Section 6.1, apart from the gap opening, another important aspect of an electrothermal microgripper designed for the manipulation of biological cells is the temperature developed at the cell gripping zone. Figure 15 compares the temperature and gap opening values at the microgripper arm tips for all the design variants at actuation voltages of 10 V and 12 V. Experimental temperature measurements of the actuated microgrippers by means of microthermal microscopy are being proposed for future work in order to verify these developed temperatures obtained numerically. It can be observed that sufficient gap openings (≥8 μm) for handling an RBC are achieved at an actuation voltage that is greater than or equal to 12 V for both the design variants with and without metal deposition on the cold arm. However, it can also be noticed that, with such an actuation voltage, the temperature at the arm tips ranges between 435 K–470 K for the different microgripper structures, posing a risk of damage to the handled RBCs. Figure 15 shows that the optimal combination of gap opening and temperature at the arm tips, in terms of a gap opening that is greater than or equal to 8 μm and a minimum tip temperature, is obtained from a microgripper structure with a cold arm width of 70 μm. However, the temperature at the tip in this case (435 K) still needs to be further limited not to exceed 400 K for the microgripper to be suitable for biomedical applications. Since the microgripper material (SCS) with its characteristic thermal properties is established by the chosen fabrication process (SOIMUMPs™), it can be concluded that a modification in the design of the microgripper arms is required in order to overcome the tip temperature limitation of these SOIMUMPs™ microgripper structures. The geometry of the gripping arms can be modified such that their resistance is increased, thus restraining heat conduction through the microgripper arm to the tip. Another possibility is to add a number of fins to the microgripper arm design. Such fins will be in the form of surfaces that extend from the microgripper arms, and their purpose will be to increase the surface area of the gripping arms, resulting in increased convective heat transfer to the surroundings and in a consequent reduction in the temperature developed at the arm tips.

## 7. Conclusions

This paper has presented a number of design variants of a horizontal electrothermal MEMS microgripper that was developed for the micromanipulation and deformability study of RBCs. The different microgripper structures were achieved by varying the cold arm width (14 μm, 30 μm, 55 μm, 70 μm and 100 μm), and by depositing a metal layer, consisting of gold with a chromium adhesion promoter, on the top surface of the cold arm. These parameters were varied in order to investigate the effects of dimensional and material variation of the cold arm on the temperature difference between the hot and cold arms, and ultimately on the performance of the microgripper mainly in terms of the achieved end-effector displacement and the temperature at the cell gripping zone. Different ways of maximising this temperature difference have been presented by designing the hot arm with the smallest possible cross-sectional area, by increasing the width of the cold arm, and by depositing a metal layer on the cold arm. A commercially available fabrication technology known as SOIMUMPs™ was used to fabricate the microgripper designs studied in this work. All structures were made of SCS and had a thickness of 25 μm.

The electrothermomechanical performance, specifically the temperature distribution and the lateral deflection at the microgripper arm tips, were investigated and compared for the different microgripper designs. Steady-state numerical simulations were performed with the commercial software package CoventorWare${}^{®}$ on the ten microgripper design variants to investigate the performance of the microgrippers under electrothermal actuation. The numerical models also took into account the temperature dependency of the thermal conductivity, the coefficient of thermal expansion and the electrical conductivity of SCS, as well as of the coefficient of thermal expansion of the chromium/gold layer. Failure to include the temperature dependancy of the electrical conductivity property of SCS in the numerical model resulted in errors as high as 52% and 34% in the resistance and power dissipation values, respectively.

It could be observed that the increase in cold arm width in the case of the design variants of the SCS microgripper structure without metal deposition on the cold arm resulted in a larger temperature difference between the hot and cold arms and in an increase in the achieved gap opening at the microgripper arm tips. Moreover, for a certain cold arm width, metal deposition on the cold arm further increased the temperature difference between the hot and cold arms. It could, however, be noted that, in the case of the design variants with metal deposition on the cold arm, the benefit of depositing metal on the cold arm to maximise the resulting temperature difference decreased as the cold arm width increased, resulting in similar gap openings achieved for the different cold arm widths. This is due to the resulting reduction in both the hot arm and cold arm temperatures with increasing cold arm width in the case of the design variants with metal deposition on the cold arm, causing a much smaller effective temperature difference achieved between the hot and cold arms.

All fabricated microgripper structures were actuated under atmospheric pressure and the achieved displacement at the arm tips was investigated via optical microscopy studies. Good agreement was achieved between the tip displacement results from the actuation testing and the numerical predictions. The temperature at the cell gripping zone was also studied numerically and compared with the gap opening for each design variant. It could be observed that the optimal combination of gap opening and temperature at the arm tips for RBC manipulation was obtained from a microgripper structure with a cold arm width of 70 μm. However, the temperature at the tip in this case still needs to be further optimised to ensure the safe manipulation of RBCs with a SOIMUMPs™ microgripper design. Moreover, other possible applications of the designed microgripper whereby the temperature at the tip is not a limitation are pick-and-place tasks that are widespread operations within the industrial sector, and that may include the assembly of rigid micromechanical parts as well as the handling of microcomponents for testing and characterisation purposes.

Future work will thus focus on the enhancement of the presented microgripper design to include force feedback, on the modification of the microgripper arm geometry to limit the temperature developed at the cell gripping zone for biomedical applications as well as on the attainment of thermal experimental results via thermal microscopy studies. All these factors will build on the current work in order to futher optimise the SOIMUMPs™ microgripper structure for the successful deformability study of RBCs.

## Figures and Tables

**Table 1:** Length and width dimensions of the studied microgripper designs. These dimensions are illustrated in Figure 2.

Parameter	Value (μm)
Length of hot arm, $L_{h}$	200
Length of cold arm, $L_{c}$	154
Length of flexure, $L_{f}$	46
Length of connector, $L_{g}$	3
Length of gripping arm, $L_{a}$	203
Width of hot arm, $w_{h}$	3
Width of cold arm, $w_{c}$	14, 30, 55, 70, 100
Width of flexure, $w_{f}$	3
Width of gripping arm, $w_{a}$	6

**Table 2:** Comparison of measured and simulated resistance and power dissipation values, with the simulated values calculated assuming temperature-independent electrical conductivity properties (i.e., constant R). The given values are for a microgripper design with a non-metallised cold arm having a width of 55 μm.

V (V)	Measured Data			Simulated Data			Error	
	I (mA)	R (kΩ)	P (mW)	I (mA)	R (kΩ)	P (mW)	R (%)	P (%)
0.5	0.38	1.33	0.19	0.39	1.30	0.19	2.64	2.57
1.0	0.73	1.38	0.73	0.77	1.30	0.77	6.47	6.07
1.5	1.10	1.36	1.65	1.16	1.30	1.74	5.06	4.82
2.0	1.47	1.37	2.93	1.54	1.30	3.09	5.38	5.10
2.5	1.82	1.37	4.55	1.93	1.30	4.82	6.03	5.68
3.0	2.17	1.38	6.50	2.32	1.30	6.95	6.81	6.37
3.5	2.51	1.40	8.77	2.70	1.30	9.46	7.76	7.20
4.0	2.84	1.41	11.35	3.09	1.30	12.35	8.83	8.11
4.5	3.16	1.43	14.20	3.47	1.30	15.63	10.06	9.14
5.0	3.43	1.46	17.15	3.86	1.30	19.30	12.52	11.13
5.5	3.72	1.48	20.48	4.25	1.30	23.35	14.03	12.30
6.0	4.02	1.49	24.12	4.63	1.30	27.79	15.20	13.20
6.5	4.21	1.55	27.33	5.02	1.30	32.61	19.31	16.19
7.0	4.35	1.61	30.44	5.40	1.30	37.82	24.27	19.53
7.5	4.68	1.60	35.09	5.79	1.30	43.42	23.75	19.19
8.0	4.92	1.63	39.34	6.17	1.30	49.40	25.58	20.37
8.5	5.06	1.68	42.97	6.56	1.30	55.77	29.79	22.95
9.0	5.27	1.71	47.39	6.95	1.30	62.52	31.94	24.21
9.5	5.44	1.75	51.66	7.33	1.30	69.66	34.84	25.84
10.0	5.60	1.79	55.97	7.72	1.30	77.19	37.91	27.49
10.5	5.73	1.83	60.17	8.10	1.30	85.10	41.44	29.30
11.0	5.85	1.88	64.35	8.49	1.30	93.40	45.14	31.10
11.5	6.00	1.92	69.00	8.88	1.30	102.08	47.94	32.41
12.0	6.10	1.97	73.20	9.26	1.30	111.15	51.84	34.14

**Table 3:** Material properties for the silicon-on-insulator multi-user MEMS process (SOIMUMPs)™ as extracted from the CoventorWare${}^{®}$ Materials Library [31]. Unless otherwise specified, these material properties are defined at 300 K.

Property	SOI	Pad-Metal
Density (g/(cm)${}^{3}$)	2.50	19.30
Young’s modulus, *E* (GPa)	$E_{x}$ = $E_{y}$ = 169	57
	$E_{z}$ = 130	
Shear modulus, *G* (GPa)	$G_{yz}$ = $G_{zx}$ = 79.6	-
	$G_{xy}$ = 50.9	
Poisson’s ratio, ν	$\nu_{yz}$ = 0.36, $\nu_{zx}$ = 0.29	0.35
	$\nu_{xy}$ = 0.064	
Specific heat capacity, *c* (J/kgK)	712	128.7
Thermal expansion coefficient, α (K${}^{-1}$)	Refer to Figure 5	Refer to Figure 6
Thermal conductivity, *k* (W/mK)	Refer to Figure 5	297
Electrical conductivity (S/mm)	Refer to Figure 5	3.496 × 10${}^{4}$
